# Dog Owner Perceptions of Veterinary Handling Techniques

**DOI:** 10.3390/ani12111387

**Published:** 2022-05-27

**Authors:** Amber Diane Carroll, Alissa Cisneros, Hannah Porter, Carly Moody, Anastasia Chiara Stellato

**Affiliations:** 1 Department of Animal and Food Sciences, Texas Tech University, 2500 Broadway, Lubbock, TX 79409, USA; amber.d.carroll@ttu.edu (A.D.C.); alissa.cisneros@ttu.edu (A.C.); hannah.porter@ttu.edu (H.P.); 2 Department of Animal Science, University of California, Davis, One Shields Ave., Davis, CA 95616, USA; cmoody@ucdavis.edu

**Keywords:** dog, handling, veterinary clinic, owner perspectives

## Abstract

**Simple Summary:**

Improving the veterinary experience for domestic dogs and their owners is important to promote canine welfare and owner compliance with routine veterinary care. Incorporating dog owner perspectives on portions of the veterinary appointment can help increase dog owner satisfaction and clinic visits. Thus, an online survey was distributed to current dog owners residing in Canada and the United States, to assess owner agreement towards 13 handling techniques used on dogs during routine veterinary appointments, when the participants’ dog was calm, fearful, or aggressive. We also assessed the influence of participant’s pet attachment and demographic information on owner agreement towards the handling techniques. Participants (N = 1176) generally disagreed with the use of more restrictive techniques and agreed with less restrictive techniques, regardless of dog behavior. We found that dog owners preferred full body restraint used on fearful dogs if they had previous veterinary experience or were male; whereas dog owners preferred minimal restraint used on fearful dogs if they had a stronger attachment to their pets or if they did not have previous veterinary experience. As owner perspectives align with current handling recommendations, we encourage veterinarians to incorporate owner perspectives to help improve dog and owner experiences during routine veterinary care.

**Abstract:**

Veterinary care can be a source of stress for domestic dogs and their owners. If a dog owner is not satisfied with the veterinary experience, this may reduce the frequency of veterinary visits and negatively impact a dog’s health and welfare. Allowing dog owners to offer their perspectives on aspects of the veterinary appointment may help improve owner satisfaction. We assessed owner agreement towards 13 recommended handling techniques used on dogs during routine veterinary appointments, when the participants’ dog was calm, fearful, or aggressive. An online cross-sectional survey targeting current dog owners, residing in Canada and the United States, was used to examine the influence of participant’s pet attachment (using the Lexington Attachment to Pets Scale (LAPS)) and demographic information (age, gender, experience working in the veterinary field) on owner agreement towards the handling techniques. The majority of participants (N = 1176) disagreed with higher restraint techniques (e.g., full body restraint, muzzle hold) and tools (e.g., dog mask), and agreed with lower restraint techniques (e.g., minimal restraint) regardless of dog demeanor. Logistic regression models revealed that for medium/large dog owners, having previous veterinary work experience resulted in lower agreement with the use of minimal restraint (*p* < 0.0001) and higher agreement with the use of full body restraint on fearful dogs (*p* = 0.01). Small dog owners were more likely to agree with the use of minimal restraint on fearful dogs if they had a higher pet attachment score (*p* < 0.001), and were more likely to agree with full body restraint if they had previous veterinary work experience (*p* < 0.0001) or were male (*p* = 0.02). Owner perspectives align with current handling recommendations and provide further support for the use of low stress handling methods to improve owner satisfaction and dog welfare during routine veterinary care.

## 1. Introduction

Veterinary care, though a necessary part of animal care, can be a source of stress for domestic dogs. For example, when assessing the behavior of dogs entering a veterinary clinic, 60% were apprehensive and unwilling to enter [1], and when assessing behaviors across various stages of a veterinary exam, approximately 78% of dogs were reported to be fearful on the examination table based on displays of reduced posture [2]. In observing their dog’s discomfort, owners may also experience stress during veterinary appointments, as previous research detected an association between dogs showing fear and aggression in veterinary clinics and owner reports of being nervous during these visits [3]. If a dog owner is not satisfied with their experience at the veterinary clinic, frequency of veterinary visits may reduce, as a report identified that 22% of dog owners said they would visit the clinic more often if their dog was less stressed [4]. In addition, improving the relationship between the owner and veterinarian can also support clinic visits, as previous research observed that a stronger bond between pet owners and veterinarians resulted in improved compliance [5]. Maintaining a positive relationship between pet owners and veterinary staff can have a positive impact on the care an animal receives. Previous research indicates that a long-term relationship between veterinarians and pet owners is strongly associated with an owner’s satisfaction level and having a strong relationship may make an owner more forgiving of a less than positive experience [6].

Approaches to improve dog and therefore owner veterinary clinic experiences include the use of low stress handling techniques [7,8,9,10], completion of the examination in a comfortable location for the dog [10], use of chemical restraint [11], and provision of treats [12]. Studies have shown that despite making changes to the physical [13] and social [14] veterinary environment, indicators of fear (avoidance, escape, reduced posture, lip licking) persist as a result of the handling used during the physical portion of the examination. Though there is a lack of evidence on the influence of veterinary handling techniques on dogs, research has revealed that certain handling can be perceived as threatening for some dogs, as when certain areas of the body are touched (i.e., head, shoulder, under the neck) dogs can respond with fear-related behaviors (e.g., avoidance, lip licking) [15].

Current recommendations for handling during routine appointments and procedures have been detailed by Dr. Sophia Yin in the book, “Low Stress Handling, Restraint, and Behavior Modification for Dogs and Cats” [10]. These handling techniques range from minimal to full body restraint to reduce veterinary-related stress in dogs as well as the risk of injury to veterinary staff. These techniques are often applied to aid in the performance of certain procedures and to ensure handler safely by controlling the movement of the animal. For example, head restraint or full body restraint can be used for venipuncture, and muzzle holds can be used for blood draw from the jugular [10]. During a veterinary appointment, low stress handling involves using appropriate handling techniques based on assessments of dog behavior, where the level of restraint increases if minimal restraints does not allow for the completion of the exam/procedure or if there is a safety risk to veterinary staff. By applying appropriate handling techniques based on dog behavior, it is suggested that the risk of aggression may be minimized [16]. However, more restrictive handling may be opted by veterinary staff if they are not comfortable proceeding without the application of a tool or a more restrictive method. There is little to no scientific evidence to support best-practice dog handling recommendations, as such current recommendations are based on anecdotal evidence. Despite a lack of research on how often these low stress handling techniques are used, or the behavioral and physiological effects of these techniques on dogs, current recommendations suggest that these methods are designed to accelerate the examination, reduce dog and handler stress, and improve safety and owner satisfaction [7].

To further improve the dog and owner experience, it is therefore important to determine dog owner perceptions of recommended handling techniques that are applied during a routine veterinary appointment. As pet owners are known to value the opportunity to provide their feedback [17], providing dog owners with the opportunity to offer their perspectives on dog handling and restraint techniques may help improve client satisfaction, strengthen the client–veterinarian relationship, and support owner motivation to bring their dog to the veterinary clinic for regular examinations. A similar study examined owner attitudes towards feline handling techniques during routine veterinary appointments and detected that cat owners preferred the less restrictive techniques regardless of the cat’s behavior, and were more likely to agree with lower restraint techniques if they reported having a stronger pet attachment [18]. However, this study also reported owner preference for the use of gloves when the cat was fearful and aggressive [18]. Since a strong pet attachment and understanding of their animal’s behavior may not always be associated with preference towards recommended handling techniques, veterinarians are encouraged to inform pet owners on current best practices.

Using an online survey, we aimed to identify owner perspectives on canine veterinary handling techniques used during routine examinations and procedures. Further, we aimed to explore how attitudes towards handling techniques differ with the degree of pet attachment and dog behavior. It is predicted that dog owners with a higher level of pet attachment would have more disagreement with more restrictive handling techniques.

## 2. Materials and Methods

### 2.1. Questionnaire

To capture owner perspectives on veterinary canine handling techniques, an online survey was created and distributed through Qualtrics^®^ (Qualtrics Software Company, Provo, UT, USA). Participants were recruited using snowball sampling through various social media platforms, including Facebook and Twitter. This sampling method relies on referrals by encouraging participants to share the advertisement to capture groups that may be more difficult to reach [19]. To be eligible to participate, participants were required to be at least 18 years of age, residents of Canada or the United States of America, and the current primary caregiver of at least one dog (e.g., primary care and financial responsibilities). The survey collected the following information: (1) veterinary care and dog ownership information, (2) handling techniques and pet attachment, and (3) owner demographics (Supplementary Information, File S1). To reduce selection bias, those who own more than one dog were asked to answer all relevant questions for the dog with the name that starts with a letter nearest to the start of the alphabet. This study was approved by the Institutional Review Boards (IRB#2021-448) at Texas Tech University in Lubbock, Texas, United States. Informed consent was provided on the first page of the survey, and no identifiable information was collected.

Participants were presented with visual and written descriptions of 13 different handling techniques based on techniques represented in Dr. Sophia Yin’s book, “Low Stress Handling, Restraint, and Behavior Modification for Dogs and Cats” [10] and recommended within other peer-reviewed literature [7,20]. The recommended handling techniques selected for use in the survey included: muzzles, mask (material placed over the eyes and clipped behind the head), towels (towel wrapped around the neck), Elizabethan collars (cone shaped collar placed around the head), head restraint (neck secured by holding the head against handlers’ body), muzzle hold (hands holding the mouth shut), minimal restraint (hands placed on the shoulders), and full body restraint (entire body held down on its side). For the full list of techniques used and detailed descriptions see Supplementary Information, File S1.

As handling techniques vary by dog size, dog owners were shown the respective 13 handling techniques based on their report of their dog’s size, either large/medium (≥35 lbs) or small (≤35 lbs), and therefore only reported their perspectives on the handling images associated with their dog’s size. Techniques were identical differing only in minor adjustments to execution (e.g., full body restraint for a large dog involved two handlers vs. one handler for a small dog). To prevent bias among participants, all techniques were portrayed through simple black and white line drawings with only the minimum detail necessary to portray each technique. A large dog was represented by a Labrador Retriever and a small dog was represented by a Chihuahua, as these breeds are common and easily identifiable. The handlers shown were drawn with minimal facial features to prevent additional bias.

Participants were asked to rate their level of agreement with each technique when their dog was calm, fearful, or aggressive, using a 5-point Likert scale (Strongly agree, Somewhat agree, Neither agree/disagree, Somewhat disagree, Strongly disagree). To ensure consistency and reduce information bias, they were given definitions of calm (relaxed, with no fear-related or aggressive behaviors), fearful (lowered posture, ears back, tail tucked, whimpering or whining, shaking or trembling, attempts to hide or escape), and aggressive behavior (baring teeth, attempting to bite, growling, lunging). Participants were also asked about their level of agreement regarding four possible veterinary clinic scenarios that were adapted from a similar study exploring cat owner perspectives on handling techniques [18]. Lexington Attachment to Pets Scale (LAPS) was used to capture the level of attachment owners have with their dog and determine whether their attachment influences owner agreement towards the veterinary handling techniques. The LAPS, which is reported to have high content validity and internal consistency (coefficient alpha = 0.93), consists of 23 statements focusing on strong pet attachment [21]. It uses a 4-point Likert scale ranging from Strongly Agree to Strongly Disagree, including a “no response” option.

### 2.2. Data Analysis

All analyses were performed with Stata Statistical Software v.17.0 (StataCorp., College Station, TX, USA).

LAPS scores were calculated by substituting the Likert agreement responses (Strongly agree, Somewhat agree, Somewhat disagree, Strongly disagree), with numerical scores of 4, 3, 2, and 1, respectively, and for negatively worded statements the order was reversed [21]. Numerical scores were then totaled to create a single LAPS score for each participant.

Logistic regression models were created to evaluate associations between owner demographics and pet attachment on agreement with the use of full body restraint and minimal restraint on fearful dogs. To account for any biases associated with dog size, the data was separated into medium/large and small dog participants, and therefore logistic regression models were created and analyzed within each group. Binomial outcomes for the logistic regression models were created by combining the categories strongly agree, somewhat agree, somewhat disagree, and strongly disagree levels, to produce ‘Agree’ and ‘Disagree’ outcome categories, respectively. Explanatory variables included in the models were: LAPS score, owner age (18–24, 25–34, 35–44, 45–54, 55–75+), gender (female, male, non-binary), previous experience working in the veterinary field, dog size (medium/large, small), and owner reports of their dog displaying fear or aggression when certain areas of their body are handled by the veterinarian. A stepwise selection process was used to create the models and model fit was assessed Bayesian Information Criteria (with a lower value preferred).

A Friedman’s test was performed to evaluate differences in the level of dog owner agreement with use of the 13 handling techniques when their dog is calm (*n* = 1176), fearful (*n* = 1174) or aggressive (*n* = 1173). Post hoc pairwise comparisons were analyzed when significant effects were detected, with a Bonferroni adjustment using a Wilcoxon test. For all tests, *p* < 0.05 was considered significant.

## 3. Results

### 3.1. Descriptives

The questionnaire was answered by a total of 1207 participants. After excluding those that did not meet inclusion criteria or did not answer questions related to their agreement to the 13 handling techniques, a total of 1176 participants remained and were included in analysis. Of the participants, most were residents of the United States (86.3%; 1015/1176) and 13.7% (161/1176) were residents of Canada. The majority of participants identified as female (74%; 870/1176), 25% (295/1176) identified as male, and 1% (8/1176) identified as non-binary. Most of the participants were between the ages of 18 to 24 (33.67%; 396/1176), 25–34 (29.3%; 345/1176), and 35–44 (18.7%; 220/1176) years of age. Regarding prior experience in the veterinary field (e.g., veterinarian, veterinary assistant, technician), 56.2% (661/1176) indicated they had no prior experience, and 43.8% (515/1176) reported having experience. Regarding dog size, 60% (706/1176) of owners reported their dog to be medium/large, and 40% (470/1176) reported their dog to be small in size. Additionally, when asked if their dog experiences fear (e.g., avoiding contact, reduced posture, whining, shaking) or aggression (e.g., barking, growling, baring teeth) when certain areas of their body are handled by the veterinarian, 55.8% (657/1176) reported no fear or aggression, and 41.6% (489/1176) reported their dog displays fear or aggression.

When participants were asked about their level of agreement regarding certain procedures when bringing their dog to the veterinarian, participants reported strong agreement with the veterinarian spending time with their dog before initiating the exam (65.7%; 773/1176; Table 1) with only 10.6% (124/1176) reporting strong agreement with veterinarian examining their dog without first spending time with them. Regarding sedation during a routine exam, 21.3% (251/1176) strongly disagreed and 22.4% (263/1176) strongly disagreed with their dog moved to treatment area to complete the examination without the owner present.

### 3.2. Owner Agreement with Canine Handling Techniques

**Figure 1:**
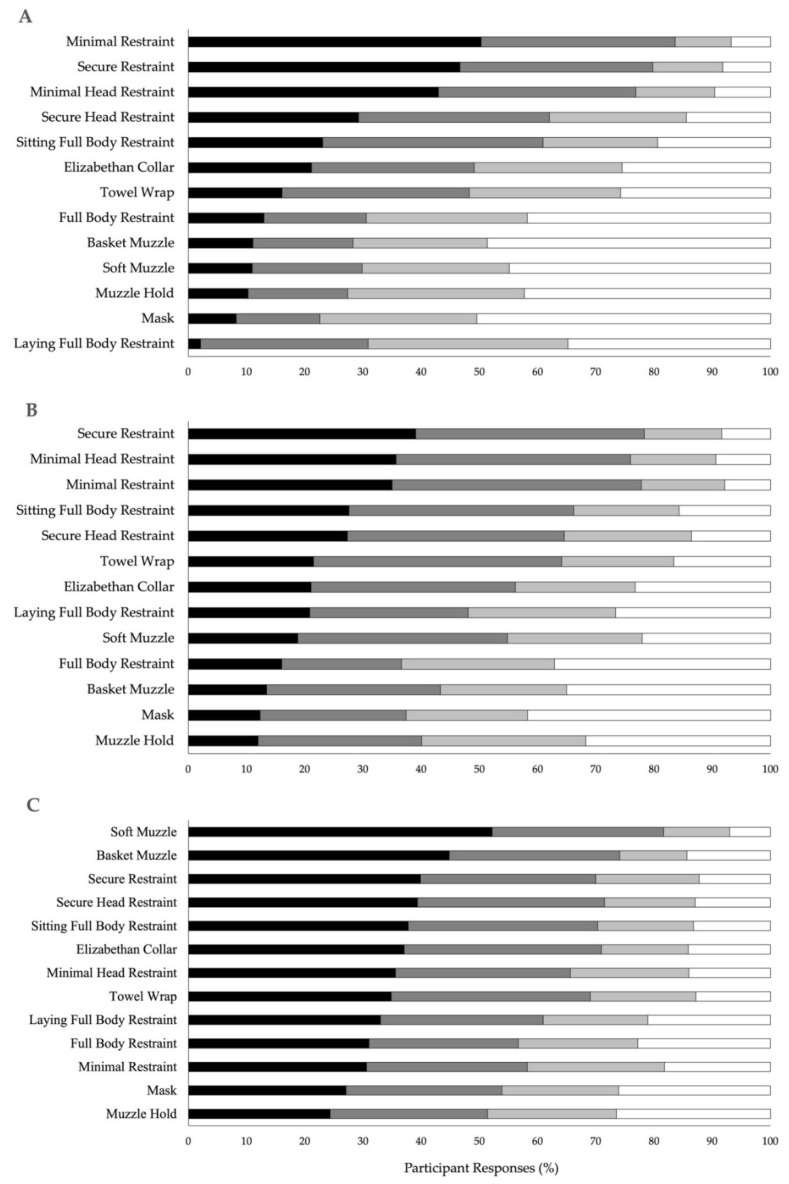
Participant’s percentage agreement with 13 different recommended handling techniques used on dogs during veterinary care when the participant’s dog is: (**A**) calm, (**B**) fearful, and (**C**) aggressive. Agreement was based on the following Likert scale responses: strongly agree (black bars), somewhat agree (dark gray), somewhat disagree (light gray), and strongly disagree (white bars). As the number of participants responding to each handling technique varied, the cumulative number of responses was <1174.

Across the 13 canine handling techniques, the level of agreement varied for calm (*p* < 0.0001; Figure 1A), fearful (*p* < 0.0001; Figure 1B), and aggressive behavior (*p* < 0.0001; Figure 1C). For each type of behavior, post hoc analysis detected significant differences in agreement between most comparisons even though a conservative correction was applied to account for multiple comparisons. Given that most of the handling techniques were significantly different, comparisons where significant differences were not observed (*p* > 0.05) are reported in Table 2.

### 3.3. Logistic Regression Results

To account for any bias associated with dog size, size was tested but not identified as a confounder, as it did not cause a 20% or greater change in a coefficient of another variable in the models. Models were analyzed separately according to dog size, and participants in each dog size group had similar demographics.

For medium/large dog owners, there were lower odds (95% confidence interval (CI)) of agreement with minimal restraint on fearful dogs if owners reported to have work experience in the veterinary field, (Odds Ratio (OR): 0.3, CI: 0.18–0.49, *p* < 0.0001). For small dog owners, agreement with minimal restraint for fearful dogs was influenced by their LAPS score, where LAPS score increased with the odds (95% CI) of agreement (OR: 1.05, CI: 1.04–1.07, *p* < 0.001). No other main effects significantly influenced owner agreement with minimal restraint.

For medium/large dog owners, there were higher odds (95% CI) of agreement with full body restraint on fearful dogs if owners reported to have previous veterinary work experience (OR: 1.8, CI: 1.14, 2.70, *p* = 0.01). For small dog owners, agreement with full body restraint was associated with owner gender (*p* = 0.02), and veterinary work experience (*p* < 0.0001). Specifically, there were higher odds of agreement if reported to be male (compared to female) (OR: 1.75, CI: 1.08, 2.82), and if reported to have previous veterinary work experience (OR: 3.56, CI: 2.02, 6.28). LAPS score was not associated with owner agreement with the use of full body restraint on fearful dogs regardless of size, and no other main effects significantly influenced owner agreement with full body restraint.

To test for dog size effects, data was combined and analyzed across both groups. Size of the dog was not a significant predictor of dog owner preference towards handling techniques.

## 4. Discussion

Owner perspectives on the application of the 13 handling techniques varied with dog demeanor. Overall, owners did not agree with techniques that involved more restraint, such as full body restraint, muzzle hold, and tools applied to the dog, such as a dog mask. Agreement with the use of more restrictive methods increased as the dog’s demeanor changed from calm to aggressive.

Dog owners showed disagreement with the use of full body restraint and laying full body restraint regardless of dog demeanor; however, if the dog was aggressive there was less agreement with minimal restraint. Current recommendations suggest using low stress handling, such as minimal restraint, while higher degrees of restraint used only if necessary and based on the dog’s behavior. The owner perspectives from the current study therefore align with these recommendations to apply higher degrees of restraint when needed to safely complete the examination. Most of the participants disagreed with muzzling techniques that required higher degrees of restraint applied to the dog’s mouth (i.e., muzzle hold) regardless of dog demeanor. The confining appearance and lack of owner familiarity with the mask may have driven their disagreement with its use, though anecdotal reports suggest that the tool may be helpful for dogs with aggression issues through reducing visual stimuli that may induce aggression [7]. Dog owners displayed agreement with muzzling tools, such as soft muzzle and basket muzzle, when the dog was aggressive. This aligns with current research and recommendations to use these techniques when dogs display signs of aggression to reduce the risk of injury to veterinary staff and allow the completion of the examination [7]. For example, a previous survey exploring owner reports on muzzle use and the effects on their dogs, suggests that despite owner reports of negative behavior changes upon muzzle application (distressed, anxious, insecure, etc.), they reported these changes to be advantageous in a veterinary setting [22]. Similarly, in exploring the influence of muzzles on dog stress levels, results from previous studies revealed more reduced postures (head, tail, ears) and inactivity [23]. Further research is needed to explore the influence of various types of handling techniques and tools on dog behavioral and physiological stress levels.

Owner reported dog attachment influenced their agreement with handling methods, as small dog owners were more likely to agree with the use of minimal restraint techniques if they had a higher attachment score. Recent research exploring pet attachment of small dog owners identified three forms of attachment, simple cohabitation, close companion, and strong bond [24]. It is unclear why in the current study pet attachment predicted agreement with handling techniques for small dog owners and not large dog owners. Though previous research has explored the differences between small and large dog owners [25,26], to the authors knowledge it is unknown as to whether small dog owners have a stronger pet attachment than large dog owners. Current results align with a similar study that explored cat owner perspectives on veterinary handling techniques, as cat owners with a high pet attachment score were more likely to disagree with full body restraint with scruff being used on fearful cats [18]. Further, previous research indicates that owners are more likely to seek preventative care if they have a stronger pet attachment [5]. The use of minimal restraint should therefore be encouraged for use on dogs since owners that seek veterinary care are more likely to agree with this technique. Taking these owner perspectives and preferences into account when determining how to best handle a dog during an examination may improve client satisfaction, resulting in more consistent veterinary care and a better veterinary experience for dogs and their owners.

Prior experience working in the veterinary field was associated with less agreement with the use of minimal restraint and more agreement with full body restraint on fearful dogs, regardless of dog size. These results suggest that dog owner perceptions on restraint depends if they have veterinary-related work experience and is possibly capturing the different perspectives on the rationale for using these techniques. For instance, owners with experience working in the veterinary field may agree with the use of a higher level of restraint on fearful dogs as these methods are often used to restrict the animal’s movement to ensure the safety of the personnel handling the dog and to ensure completion of the exam. Though previous research reveals that 75% of veterinarians surveyed reported having been bitten by dogs [27], and 71% reported having an occupational injury, with most injuries caused by bites and scratches from dogs and cats [28], the efficacy of these restraint methods in reducing the risk to injury has yet to be evaluated. This contrasts with the low stress handling philosophy, where the use of restrictive handling techniques is not recommended as it is more likely to increase fear and thus the potential for aggression [20]. Further, dog owners without experience working in the veterinary field were more likely to agree with the use of lower restraint methods, potentially suggesting their perspective on prioritizing the comfort of the dog being handled. Though more restrictive methods are not recommended to be used on fearful or aggressive dogs, it is possible that dog owner preferences could change if explicitly made aware that veterinary staff may opt to use these methods to increase personal safety. These results highlight the needs for further education on the utility of and benefits associated with low stress handling during routine veterinary care. Further research is also needed to understand the differences in handling perspectives between dog owners in the veterinary field and dog owners that are not.

Our results also reveal that male, small dog owners were more likely to agree with the use of full body restraint on fearful dogs. Though this result could be explained by the temperament of dogs owned by men, previous research indicates that men tend to have calmer dogs than females [29], and owner gender has not been identified as a predictor for veterinary-related fear and aggression in dogs [3]. It is possible that these gender results may be linked to whether they have veterinary work experience; however, significant interactions were not detected between these variables and there was a relatively even representation of genders for those with veterinary work experience. Additional research is needed to better understand the influence of dog owner gender on improving the dog owner experience during veterinary visits.

Participants also displayed preferences for aspects of the veterinary examination surrounding interactions with the veterinarian. For instance, results suggest strong owner agreement for veterinarians spending time with the dog prior to beginning the examination, and disagreement with their dog being taken to a separate treatment area without the owner present, and having their dog sedated for the exam. These perspectives align with current recommendations to reduce veterinary-related fear in dogs during routine appointments. For instance, research examining the influence of owner presence on dog fear levels during a routine examination revealed that dogs display less fear behaviors (e.g., reduced vocalizations and heart rate), when the owner is present [14]. Additionally, it is generally recommended that a sedative or anxiolytic (e.g., trazodone) be administered prior to the appointment [7,9,20], as dogs provided with a sedative have been found to display fewer signs of stress 45 min after delivery of the medication [11]. It is possible that participants did not prefer this method, as they may perceive it as invasive or unnecessary, as it is only recommended for dogs that are highly fearful or aggressive. Although our study results highlight areas where client satisfaction and owner experience can be improved, further research is necessary to understand dog owner perspectives on routine veterinary care.

In the current study, there were a number of limitations that may influence generalizability. For example, the majority of participants were from the United States, which may affect how accurately the results can be generalized to Canadian dog owners. However, there were no significant country effects and both countries were reasonably represented due to the large sample size. Additionally, as similarly observed in other online surveys targeted to pet owners [18,30,31], the majority of participants were female, and this may influence how well the results reflect the opinions of male or non-binary dog owners. Further, as the survey relied on self-reporting of owner perspectives, it is possible that social desirability bias (owners answer questions in a way they deem to be more socially acceptable) may have been introduced. However, to minimize impacts from this bias, participant responses were not connected to any identifying information. A further limitation in using owner perspectives is based on the average dog owner not having a veterinary science education, therefore caution should be made in veterinarians incorporating owner perspectives when perspectives and current scientific evidence do not align. Additionally, recruiting dog owners via social media platforms may have introduced bias by sharing the advertisement to dog-interest groups that may have more interest in dog-related topics, and certain perspectives on dog ownership and veterinary care.

## 5. Conclusions

Owner satisfaction plays a significant role in improving compliance with veterinary care. By evaluating owner perspectives on handling techniques that can be used during examinations, the results suggest that owners prefer the use of minimal restraint techniques when the animal is calm, but their preferences change if the dog displays aggressive behavior. Furthermore, small dog owners preferred the use of minimal restraint if they had a higher pet attachment score. Educating veterinary staff on the importance of considering owner perception while safely handling a dog is vital to continuing to improve dog welfare in the veterinary environment; however, if owner perspectives and current recommendations do not align, veterinarians are encouraged to educate dog owners on current best practices.

## Figures and Tables

**Table 1:** Descriptive information (frequency, percentage) displaying participant agreement to four possible scenarios that can occur with their dog at the veterinary clinic (N = 1176).

Scenario	Strongly Agree	Somewhat Agree	Neither Agree/Disagree	Somewhat Disagree	Strongly Disagree
I am comfortable with having my dog sedated for a routine veterinary exam.	161 (13.7)	269 (22.9)	188 (16.0)	307 (26.1)	251 (21.3)
I am comfortable with the veterinarian entering the room and spending time with my dog before the exam.	773 (65.7)	185 (15.7)	129 (11.0)	87 (7.4)	2 (0.2)
I am comfortable with the veterinarian entering the room and immediately beginning to examine my dog without spending time with them first.	124 (10.6)	237 (20.2)	217 (18.5)	413 (35.2)	184 (15.7)
I am comfortable with having my dog taken to the treatment area to perform the examination, without my presence.	127 (10.8)	213 (18.1)	226 (19.2)	347 (29.5)	263 (22.4)

**Table 2:** Post hoc pair-wise comparisons of dog owner agreement with the 13 handling techniques used on calm, fearful, and aggressive dogs, revealing non-significant (*p* > 0.05) differences between owner agreement.

Handling Techniques		*p*-Value
**Calm**		
	Full body restraint vs. Soft muzzle	1.00
	Full body restraint vs. Muzzle hold	1.00
	Full body restraint vs. Basket muzzle	1.00
	Laying full body vs. Towel wrap	0.19
	Sitting full body restraint vs. Head (secure) restraint	0.07
	Head (minimal) restraint vs. Secure restraint	0.10
	Muzzle hold vs. Soft muzzle	1.00
	Basket muzzle vs. Soft muzzle	1.00
	Basket muzzle vs. Muzzle hold	1.00
	Basket muzzle vs. Mask	0.30
	E-collar vs. Towel wrap	1.00
**Fearful**		
	Full body restraint vs. Muzzle hold	1.00
	Full body restraint vs. Mask	1.00
	Full body restraint vs. Basket muzzle	1.00
	Laying full body restraint vs. Soft muzzle	1.00
	Laying full body restraint vs. E-collar	0.10
	Sitting full body restraint vs. Head (secure) restraint	1.00
	Sitting full body restraint vs. Towel wrap	1.00
	Minimal restraint vs. Secure restraint	1.00
	Head (secure) vs. Towel wrap	1.00
	Head (minimal) restraint vs. Minimal restraint	1.00
	Head (minimal) restraint vs. Secure restraint	1.00
	Basket muzzle vs. Muzzle hold	1.00
	E-collar vs. Soft muzzle	1.00
	Mask vs. Muzzle hold	0.94
**Aggressive**		
	Full body restraint vs. Minimal restraint	1.00
	Full body restraint vs. Muzzle hold	0.10
	Full body restraint vs. Laying full body restraint	0.82
	Full body restraint vs. Mask	1.00
	Laying full body restraint vs. Minimal restraint	1.00
	Laying full body restraint vs. Head (minimal) restraint	0.13
	Sitting full body restraint vs. Head (secure) restraint	1.00
	Sitting full body restraint vs. Secure restraint	1.00
	Sitting full body restraint vs. Head (minimal) restraint	1.00
	Sitting full body restraint vs. Towel wrap	1.00
	Sitting full body restraint vs. E-collar	1.00
	Sitting full body restraint vs. Basket muzzle	1.00
	Secure restraint vs. Towel wrap	1.00
	Minimal restraint vs. Mask	0.22
	Head (secure) restraint vs. Head (minimal) restraint	0.19
	Head (secure) restraint vs. Secure restraint	1.00
	Head (secure) vs. Towel wrap	1.00
	Head (minimal) restraint vs. Secure restraint	0.62
	Head (minimal) restraint vs. Towel wrap	1.00
	Mask vs. Muzzle hold	1.00
	Basket muzzle vs. Secure restraint	1.00
	Basket muzzle vs. Head (secure) restraint	1.00
	Basket muzzle vs. Towel wrap	0.09
	E-collar vs. Secure restraint	1.00
	E-collar vs. Head (secure) restraint	1.00
	E-collar vs. Head (minimal) restraint	1.00
	E-collar vs. Towel wrap	1.00
	E-collar vs. Basket muzzle	0.07

## Data Availability

The data presented in this study are available on request from the corresponding author. The data are not publicly available due to ethical restrictions.

## Supplementary Materials

The following supporting information can be downloaded at: https://www.mdpi.com/article/10.3390/ani12111387/s1, File S1: Owner perspectives on canine handling techniques distributed to current dog owners.
