# Supplementary material for: Dog Owner Perceptions of Veterinary Handling Techniques
Source: Animals (Basel). 2022 May 27;12(11):1387. doi: 10.3390/ani12111387 (PMC9179842; doi:10.3390/ani12111387)
Supplement: Supplementary file 1 [file animals-12-01387-s001.zip › animals-1684825-supplementary.pdf]

S1: Owner perspectives on canine handling techniques distributed to current dog owners.

1. Are you 18 years or older?
   - o Yes
   - o No

2. Are you the primary caregiver of at least one dog? (e.g., financial and care responsibilities)
   - o Yes
   - o No

3. In what country do you currently reside?
   - o United States
   - o Canada
   - o Other Country

4. Which province/territory do you currently live in? [drop down of Provinces]

5. Which state do you currently live in? [drop down of States]

Part I: Dog Ownership

If you have more than one dog, please respond to the following questions for the dog whose name begins with the letter closest to the beginning of the alphabet

6. When you bring your dog to the veterinarian, tell us about your agreement with each of the following statements.

I am comfortable with ...

	Strongly Agree	Somewhat Agree	Neither Agree/Disagree	Somewhat Disagree	Strongly Disagree
Having my dog sedated for a routine veterinary exam	o	o	o	o	o
The veterinarian entering the room and spending time with my dog before beginning the exam	o	o	o	o	o
The veterinarian entering the room and immediately beginning to examine my dog without spending time with them first	o	o	o	o	o

Having my dog taken to the treatment area to perform the examination, without my presence	○	○	○	○	○

7. Does your dog experience fear (e.g., avoiding contact, reduced posture, whining, shaking) or aggression (e.g., barking, growling, baring teeth) in the following situations?

	Yes	No	Don't know
When entering the clinic	○	○	○
When entering the examination room	○	○	○
When certain areas of their body are handled by the veterinarian	○	○	○

8. Thinking back to all of your dog's previous veterinary appointments, how often do you assist with handling your dog during the appointment?

- ○ Never
- ○ Rarely
- ○ Sometimes
- ○ Often
- ○ Always

9. Please select the approximate size of your dog.

- ○ Small (less than or equal to 30 lbs. / 14 kg)
- ○ Medium/large (greater than 30 lbs. / 14 kg)

Part II: Handling Techniques [will display if owner selects they have a small dog]

NOTE: Please answer the following questions with reference to the dog selected in the previous questions

Please read before continuing

For the next set of questions, you will be asked to indicate if you agree with the use of various dog handling techniques for routine examination on your dog when it is calm, fearful, and aggressive. This means you will see the same handling technique several times. Definitions will be provided.

The 3 questions will be set up in the following way:

1) When your dog is **CALM** during a routine examination, what is your level of agreement with use of the following handling techniques by veterinary staff?

2) When your dog is **FEARFUL** during a routine examination, what is your level of agreement with use of the following handling techniques by veterinary staff?

3) When your dog is **AGGRESSIVE** during a routine examination, what is your level of agreement with use of the following handling techniques by veterinary staff?

For the purposes of this study calm, fearful, and aggressive are defined in the following ways:

**CALM**	Relaxed, no signs of aggression or fear-related behaviors
**FEARFUL**	Showing fear-related behaviors, such as lowered posture, ears back, tail tucked, whimpering or whining, shaking, or trembling, attempts to hide or escape
**AGGRESSIVE**	Showing fear-related behaviors while showing aggression, such as baring teeth, attempting to bite, growling, lunging

CALM: Relaxed, no signs of aggression or fear-related behaviors

10. When your dog is **CALM** during a routine examination, what is your level of agreement with use of the following handling techniques by veterinary staff?

	Strongly Agree	Somewhat Agree	Neither Agree/Disagree	Somewhat Disagree	Strongly Disagree
**Soft muzzle**: Fabric muzzle that clips behind the ears securing the mouth shut 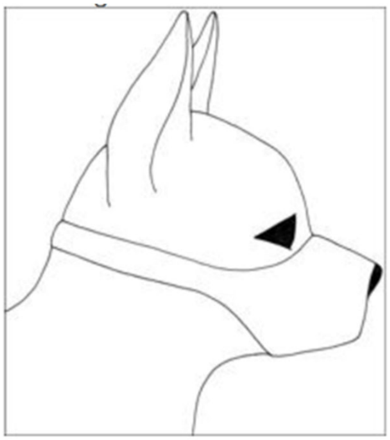	○	○	○	○	○
**Muzzle hold:** Mouth held shut with both hands on muzzle 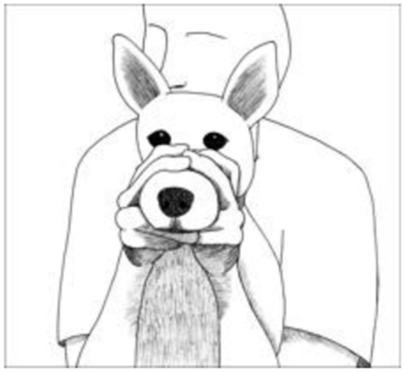	○	○	○	○	○

Towel wrap: Towel wrapped around the neck and held at the back to stabilize the head

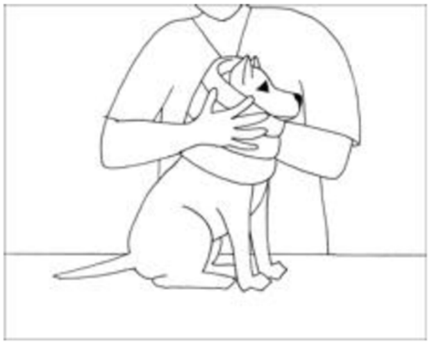

Minimal restraint: Hands are placed on each side of the dog's shoulder allowing some movement of the body and limbs

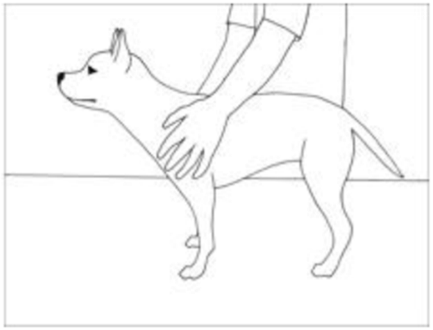

Secure restraint: Hold that secures abdomen with one hand and neck with other hand

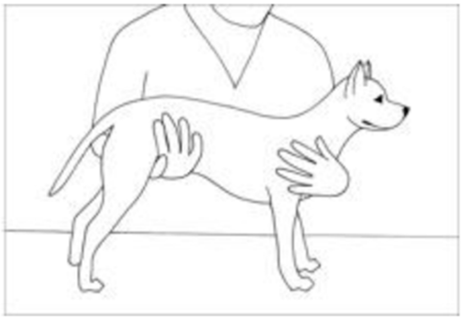

○ ○ ○ ○ ○

○ ○ ○ ○ ○

○ ○ ○ ○ ○

Head restraint (minimal): Restraint that rests hands on either side of neck with loose hold on collar

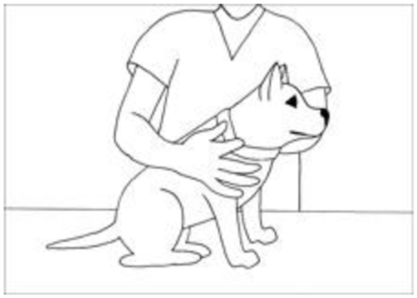

Head restraint (secure): Restraint that secures the neck by pulling head toward handlers' body with one hand and securing side with arm to prevent moving

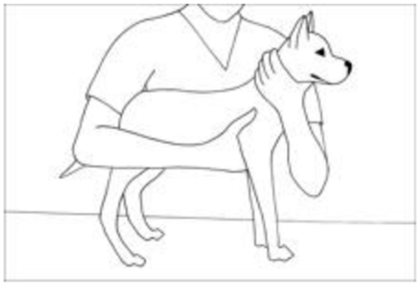

Laying full body restraint: Forward leaning motion with both hands secure around the neck to prevent standing

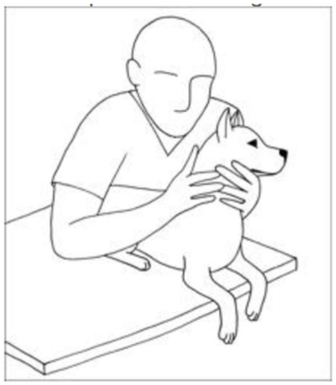

○	○	○	○	○
○	○	○	○	○
○	○	○	○	○

Full body restraint (secure): Restrain all legs using hands while dog is laying on side allowing little to no movement

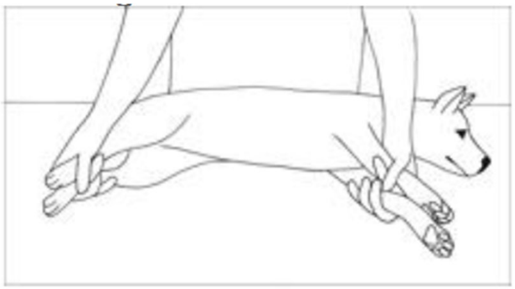

Sitting full body restraint: Restraint that secures the neck and a limb with one hand while in a sitting position to prevent moving back

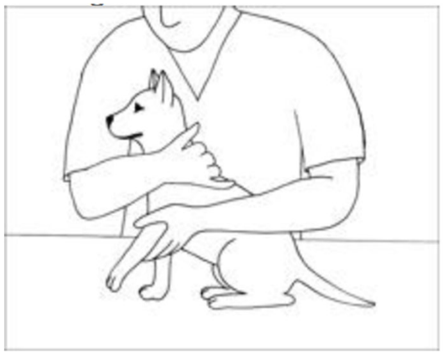

Elizabethan collar: Cone shaped collar that reduces head movement and mouth access beyond collar

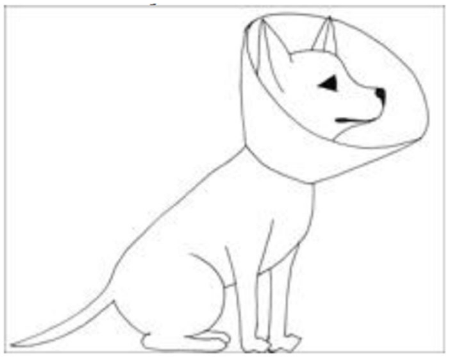

Dog mask: Mask is placed over the eyes and clipped behind the head to reduce visual stimulation

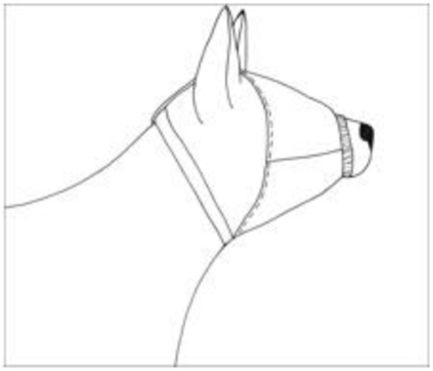

Basket muzzle: Muzzle that allows for some mouth movement but limited inside the device

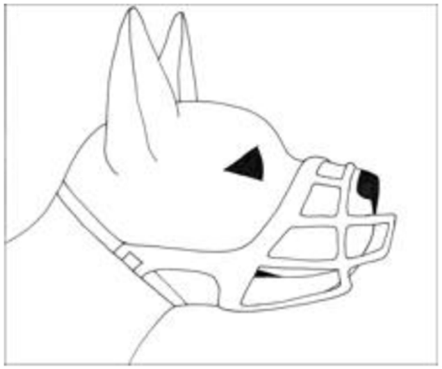

FEARFUL: Showing fear-related behaviors, such as lowered posture, ears back, tail tucked, whimpering or whining, shaking or trembling, attempts to hide or escape

11. When your dog is **FEARFUL** during a routine examination, what is your level of agreement with use of the following handling techniques by veterinary staff?

	Strongly Agree	Somewhat Agree	Neither Agree/Disagree	Somewhat Disagree	Strongly Disagree
**Soft muzzle**: Fabric muzzle that clips behind the ears securing the mouth shut 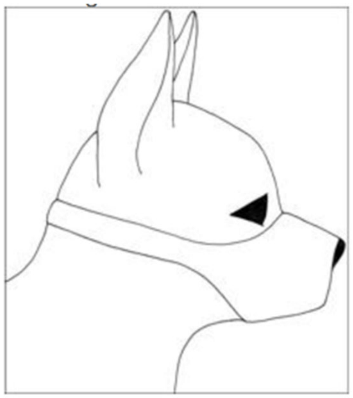	○	○	○	○	○
**Muzzle hold:** Mouth held shut with both hands on muzzle 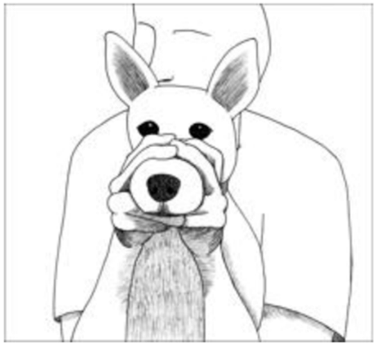	○	○	○	○	○

Towel wrap: Towel wrapped around the neck and held at the back to stabilize the head

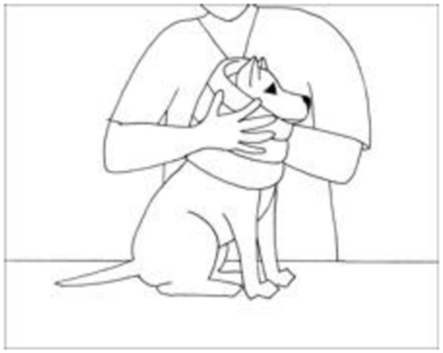

○ ○ ○ ○ ○

Minimal restraint: Hands are placed on each side of the dog's shoulder allowing some movement of the body and limbs

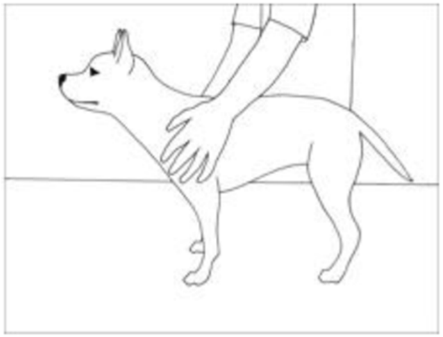

○ ○ ○ ○ ○

Secure restraint: Hold that secures abdomen with one hand and neck with other hand

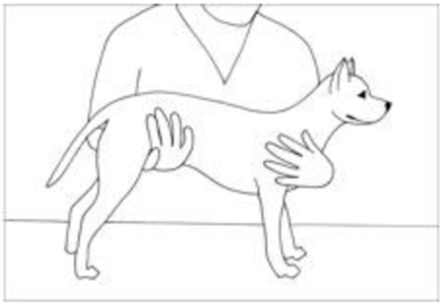

○ ○ ○ ○ ○

Head restraint (minimal): Restraint that rests hands on either side of neck with loose hold on collar

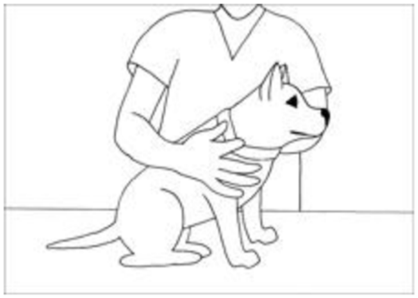

○ ○ ○ ○ ○

Head restraint (secure): Restraint that secures the neck by pulling head toward handlers' body with one hand and securing side with arm to prevent moving

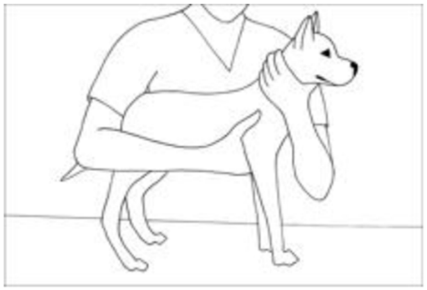

○ ○ ○ ○ ○

Laying full body restraint: Forward leaning motion with both hands secure around the neck to prevent standing

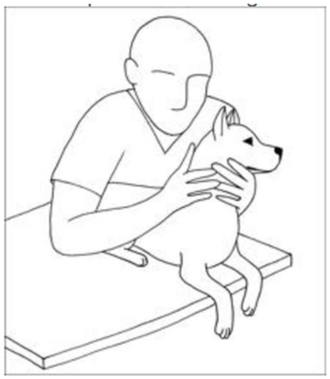

○ ○ ○ ○ ○

Full body restraint (secure): Restrain all legs using hands while dog is laying on side allowing little to no movement

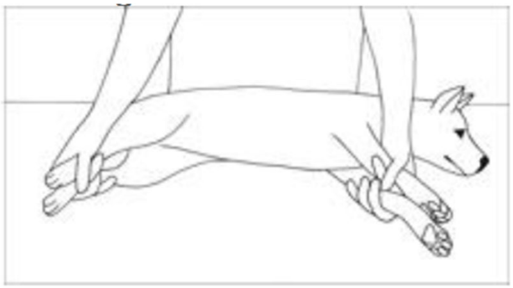

Sitting full body restraint: Restraint that secures the neck and a limb with one hand while in a sitting position to prevent moving back

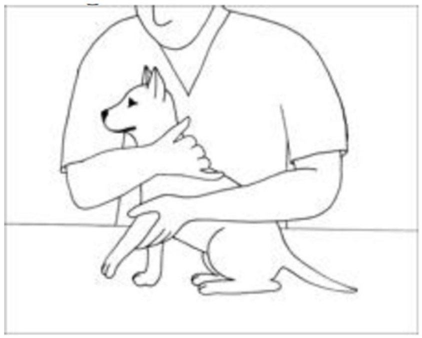

Elizabethan collar: Cone shaped collar that reduces head movement and mouth access beyond collar

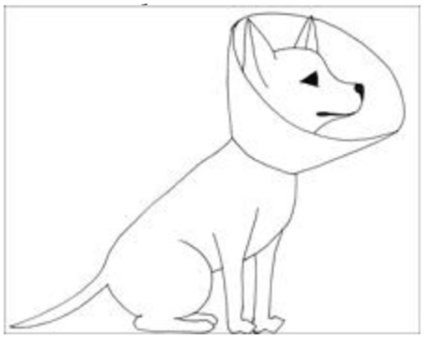

○ ○ ○ ○ ○

○ ○ ○ ○ ○

○ ○ ○ ○ ○

Dog mask: Mask is placed over the eyes and clipped behind the head to reduce visual stimulation

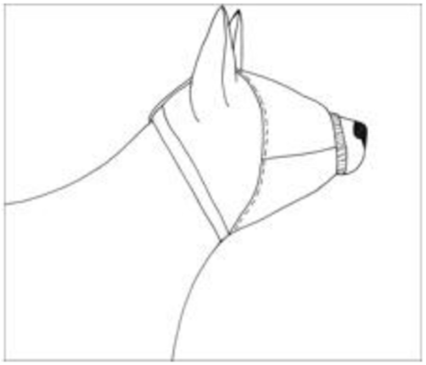

Basket muzzle: Muzzle that allows for some mouth movement but limited inside the device

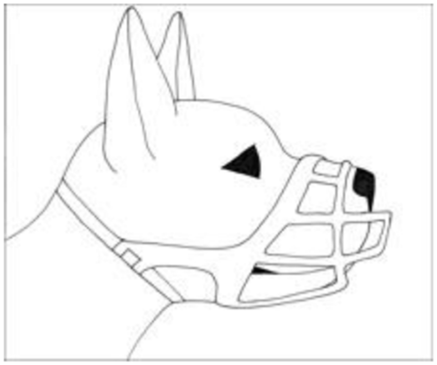

○ ○ ○ ○ ○

○ ○ ○ ○ ○

12. **AGGRESSIVE**: Showing fear-related behaviors while showing aggression, such as baring teeth, attempting to bite, growling, lunging.

When your dog is **AGGRESSIVE** during a routine examination, what is your level of agreement with use of the following handling techniques by veterinary staff?

	Strongly Agree	Somewhat Agree	Neither Agree/Disagree	Somewhat Disagree	Strongly Disagree
**Soft muzzle**: Fabric muzzle that clips behind the ears securing the mouth shut 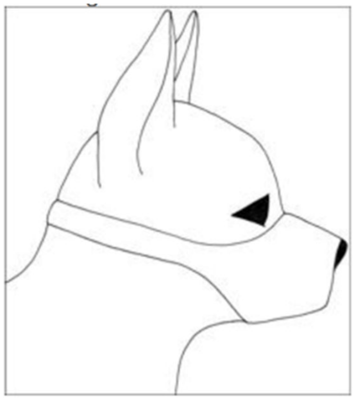	○	○	○	○	○
**Muzzle hold:** Mouth held shut with both hands on muzzle 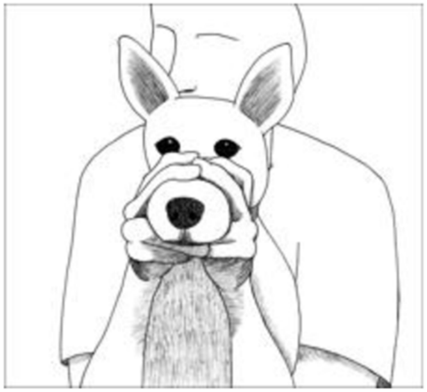	○	○	○	○	○

Towel wrap: Towel wrapped around the neck and held at the back to stabilize the head

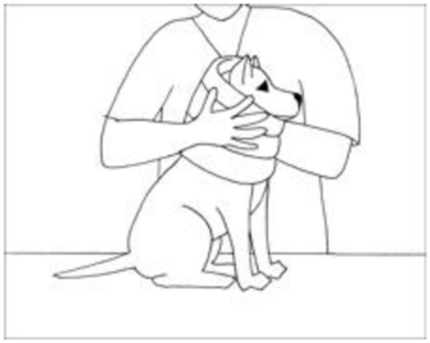

○ ○ ○ ○ ○

Minimal restraint: Hands are placed on each side of the dog's shoulder allowing some movement of the body and limbs

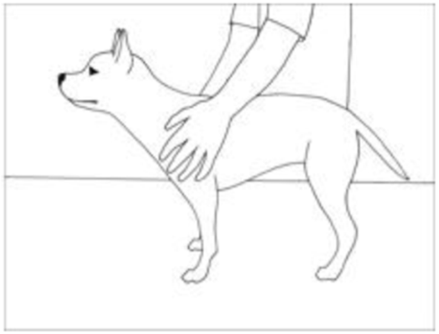

○ ○ ○ ○ ○

Secure restraint: Hold that secures abdomen with one hand and neck with other hand

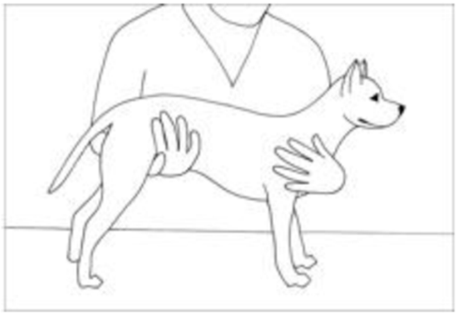

○ ○ ○ ○ ○

Head restraint (minimal): Restraint that rests hands on either side of neck with loose hold on collar

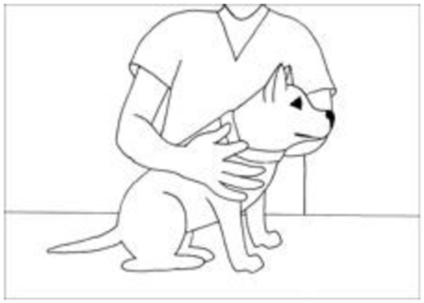

Head restraint (secure): Restraint that secures the neck by pulling head toward handlers’ body with one hand and securing side with arm to prevent moving

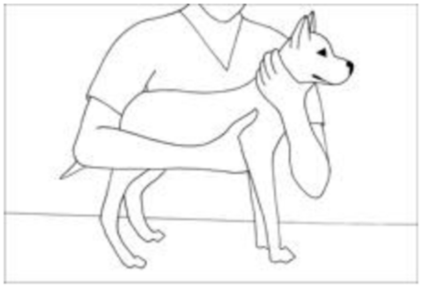

Laying full body restraint: Forward leaning motion with both hands secure around the neck to prevent standing

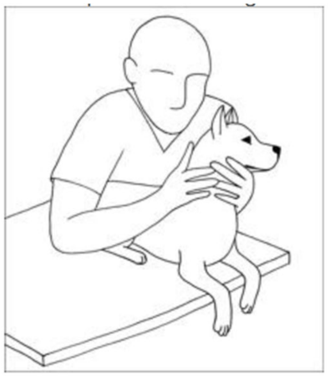

Full body restraint (secure): Restrain all legs using hands while dog is laying on side allowing little to no movement

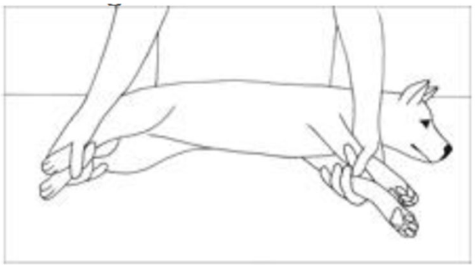

Sitting full body restraint: Restraint that secures the neck and a limb with one hand while in a sitting position to prevent moving back

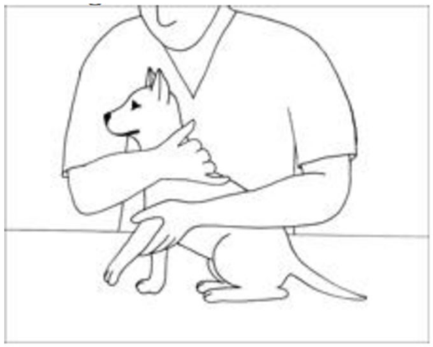

Elizabethan collar: Cone shaped collar that reduces head movement and mouth access beyond collar

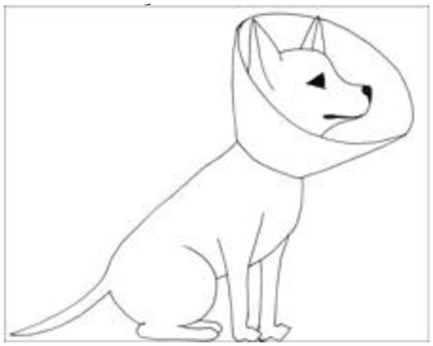

Dog mask: Mask is placed over the eyes and clipped behind the head to reduce visual stimulation

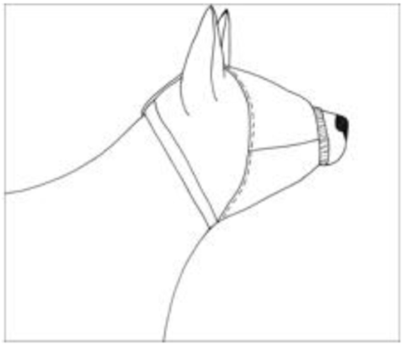

Basket muzzle: Muzzle that allows for some mouth movement but limited inside the device

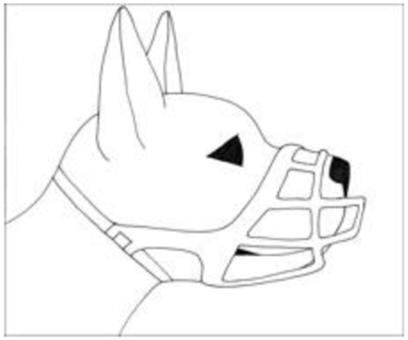

○ ○ ○ ○ ○

○ ○ ○ ○ ○

13. Please tell us your agreement with the following statements about your dog.

	Strongly Agree	Somewhat Agree	Somewhat Disagree	Strongly Disagree	No Response
My pet means more to me than any of my friends	○	○	○	○	○
Quite often I confide in my pet	○	○	○	○	○
I believe that pets should have the same rights and privileges as family members	○	○	○	○	○
I believe my pet is my best friend	○	○	○	○	○
Quite often, my feelings toward people are affected by the way they react to my pet	○	○	○	○	○
I love my pet because he/she is more loyal to me than most of the people in my life	○	○	○	○	○
I enjoy showing other people pictures of my pet	○	○	○	○	○
I think my pet is just a pet	○	○	○	○	○
I love my pet because it never judges me	○	○	○	○	○
My pet knows when I'm feeling bad	○	○	○	○	○
I often talk to other people about my pet	○	○	○	○	○
My pet understands me	○	○	○	○	○
I believe that loving my pet helps me to stay healthy	○	○	○	○	○
Pets deserve as much respect as humans do	○	○	○	○	○
My pet and I have a very close relationship	○	○	○	○	○
I would do almost anything to take care of my pet	○	○	○	○	○
I play with my pet quite often	○	○	○	○	○
I consider my pet to be a great companion	○	○	○	○	○
My pet makes me feel happy	○	○	○	○	○
I feel that my pet is a part of my family	○	○	○	○	○

I am not very attached to my pet	○	○	○	○	○
Owning a pet adds to my happiness	○	○	○	○	○
I consider my pet to be a friend	○	○	○	○	○

Part II: Handling Techniques [will display if owner selects they have a medium/large dog]

NOTE: Please answer the following questions with reference to the dog selected in the previous questions

Please read before continuing

For the next set of questions, you will be asked to indicate if you agree with the use of various dog handling techniques for routine examination on your dog when it is calm, fearful, and aggressive. This means you will see the same handling technique several times. Definitions will be provided.

The 3 questions will be set up in the following way:

1) When your dog is **CALM** during a routine examination, what is your level of agreement with use of the following handling techniques by veterinary staff?

2) When your dog is **FEARFUL** during a routine examination, what is your level of agreement with use of the following handling techniques by veterinary staff?

3) When your dog is **AGGRESSIVE** during a routine examination, what is your level of agreement with use of the following handling techniques by veterinary staff?

For the purposes of this study calm, fearful, and aggressive are defined in the following ways:

**CALM**	Relaxed, no signs of aggression or fear-related behaviors
**FEARFUL**	Showing fear-related behaviors, such as lowered posture, ears back, tail tucked, whimpering or whining, shaking or trembling, attempts to hide or escape
**AGGRESSIVE**	Showing fear-related behaviors while showing aggression, such as baring teeth, attempting to bite, growling, lunging

10. CALM: Relaxed, no signs of aggression or fear-related behaviors

When your dog is **CALM** during a routine examination, what is your level of agreement with use of the following handling techniques by veterinary staff?

	Strongly Agree	Somewhat Agree	Neither Agree/Disagree	Somewhat Disagree	Strongly Disagree
**Soft muzzle**: Fabric muzzle that clips behind the ears securing the mouth shut 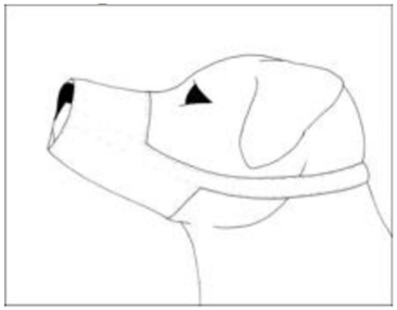	○	○	○	○	○
**Muzzle hold:** Mouth held shut with both hands on muzzle 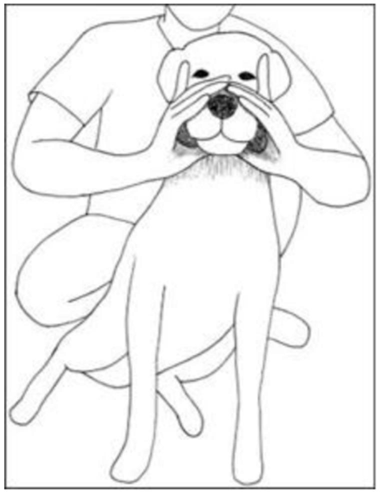	○	○	○	○	○

Towel wrap: Towel wrapped around the neck and held at the back to stabilize the head

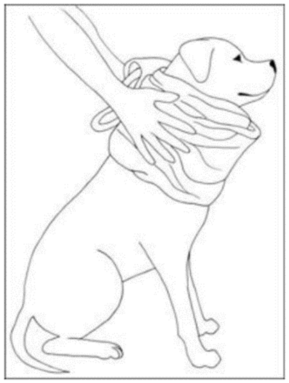

Minimal restraint: Hands are placed on each side of the dog's shoulder allowing some movement of the body and limbs

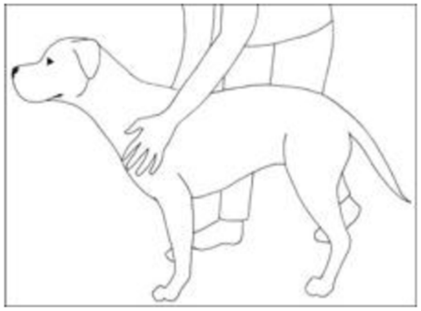

Secure restraint: Hold that secures abdomen with one hand and neck with other hand

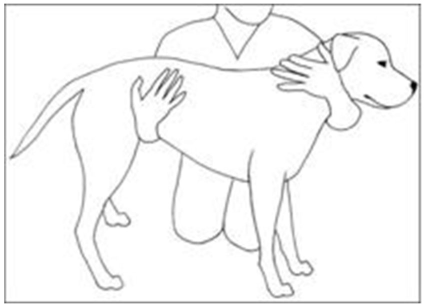

Head restraint (minimal): Restraint that rests hands on either side of neck with loose hold on collar

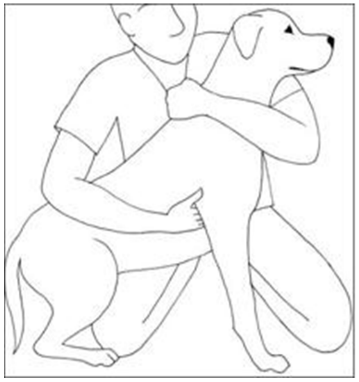

Head restraint (secure): Restraint that secures the neck by pulling head toward handlers' body with one hand and securing side with arm to prevent moving

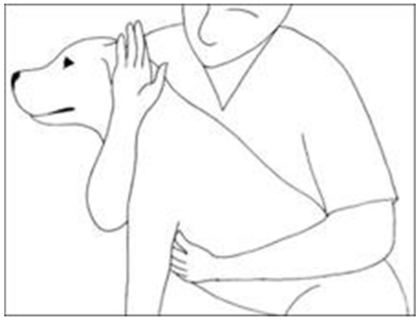

Laying full body restraint: Forward leaning motion with both hands secure around the neck to prevent standing

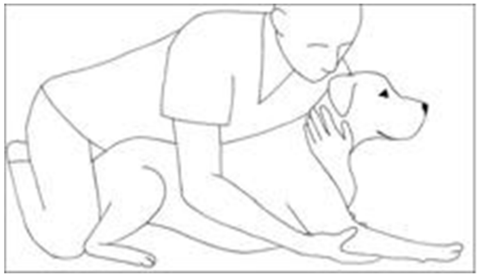

Full body restraint (secure): Restrain all legs using hands while dog is laying on side allowing little to no movement

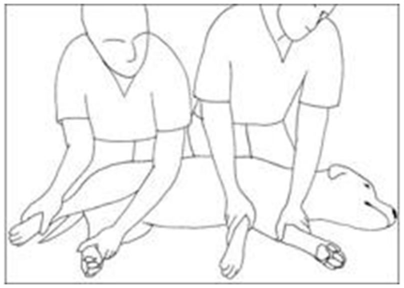

Sitting full body restraint: Restraint that secures the neck and a limb with one hand while in a sitting position to prevent moving back

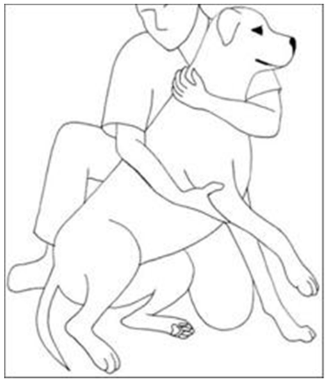

Elizabethan collar: Cone shaped collar that reduces head movement and mouth access beyond collar

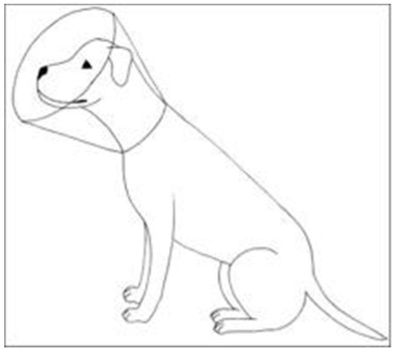

Dog mask: Mask is placed over the eyes and clipped behind the head to reduce visual stimulation

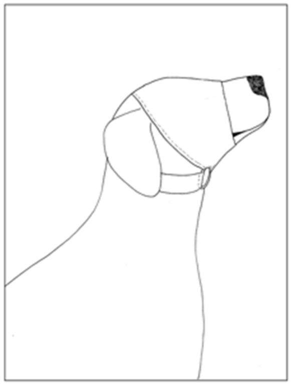

Basket muzzle: Muzzle that allows for some mouth movement but limited inside the device

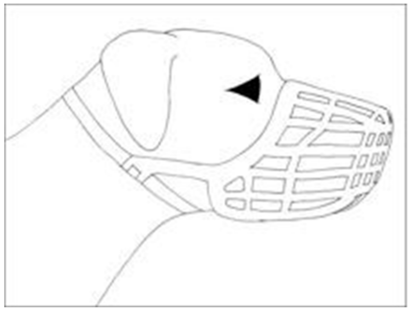

○ ○ ○ ○ ○

○ ○ ○ ○ ○

FEARFUL: Showing fear-related behaviors, such as lowered posture, ears back, tail tucked, whimpering or whining, shaking or trembling, attempts to hide or escape

11. When your dog is **FEARFUL** during a routine examination, what is your level of agreement with use of the following handling techniques by veterinary staff?

	Strongly Agree	Somewhat Agree	Neither Agree/Disagree	Somewhat Disagree	Strongly Disagree
**Soft muzzle**: Fabric muzzle that clips behind the ears securing the mouth shut 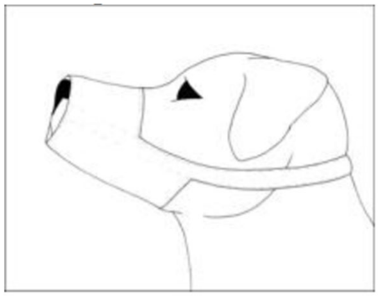	○	○	○	○	○
**Muzzle hold:** Mouth held shut with both hands on muzzle 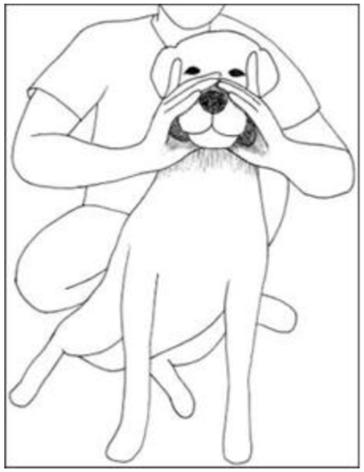	○	○	○	○	○

Towel wrap: Towel wrapped around the neck and held at the back to stabilize the head

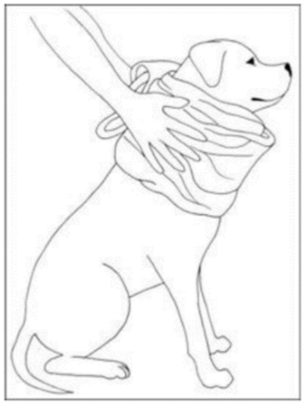

○ ○ ○ ○ ○

Minimal restraint: Hands are placed on each side of the dog's shoulder allowing some movement of the body and limbs

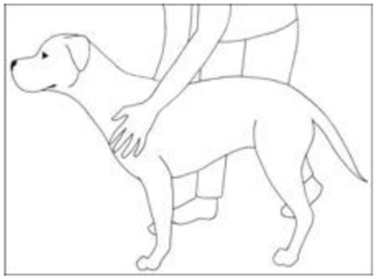

○ ○ ○ ○ ○

Secure restraint: Hold that secures abdomen with one hand and neck with other hand

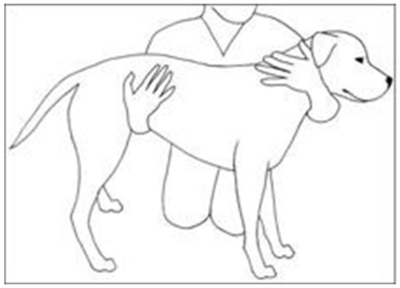

○ ○ ○ ○ ○

Head restraint (minimal): Restraint that rests hands on either side of neck with loose hold on collar

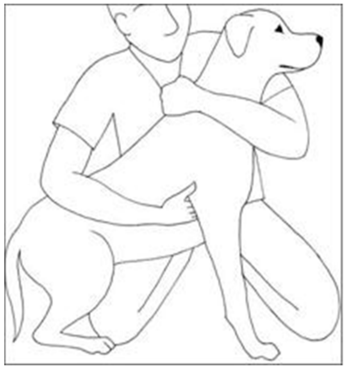

Head restraint (secure): Restraint that secures the neck by pulling head toward handlers’ body with one hand and securing side with arm to prevent moving

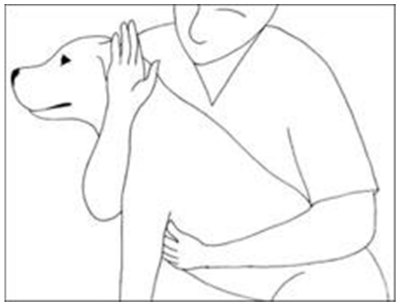

Laying full body restraint: Forward leaning motion with both hands secure around the neck to prevent standing

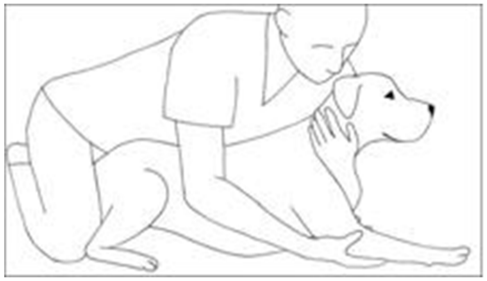

Full body restraint (secure): Restrain all legs using hands while dog is laying on side allowing little to no movement

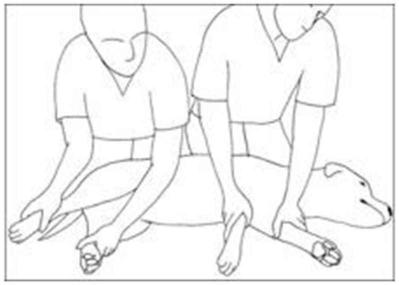

○ ○ ○ ○ ○

Sitting full body restraint: Restraint that secures the neck and a limb with one hand while in a sitting position to prevent moving back

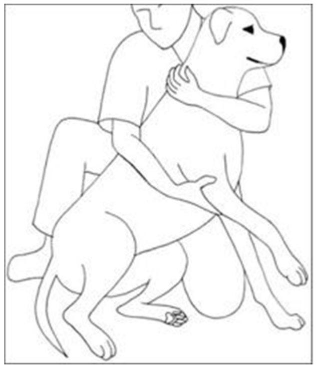

○ ○ ○ ○ ○

Elizabethan collar: Cone shaped collar that reduces head movement and mouth access beyond collar

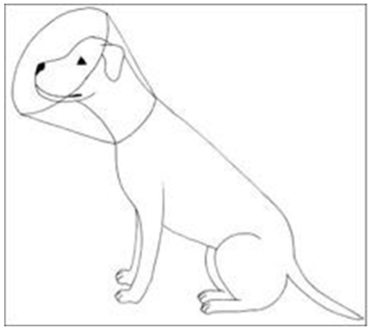

○ ○ ○ ○ ○

Dog mask: Mask is placed over the eyes and clipped behind the head to reduce visual stimulation

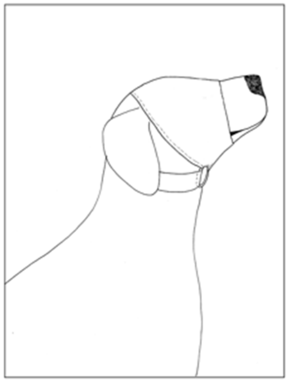

Basket muzzle: Muzzle that allows for some mouth movement but limited inside the device

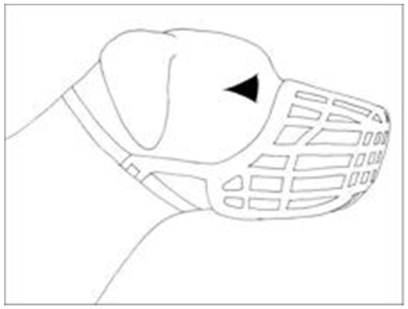

AGGRESSIVE: Showing fear-related behaviors while showing aggression, such as baring teeth, attempting to bite, growling, lunging

12. When your dog is **AGGRESSIVE** during a routine examination, what is your level of agreement with use of the following handling techniques by veterinary staff?

	Strongly Agree	Somewhat Agree	Neither Agree/Disagree	Somewhat Disagree	Strongly Disagree
**Soft muzzle**: Fabric muzzle that clips behind the ears securing the mouth shut 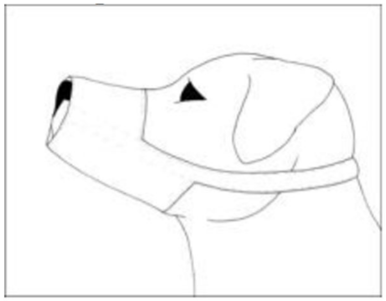	○	○	○	○	○
**Muzzle hold:** Mouth held shut with both hands on muzzle 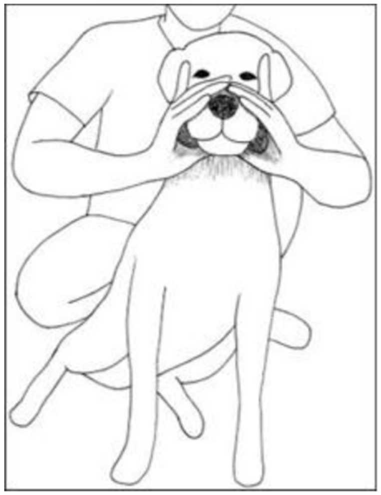	○	○	○	○	○

**Towel wrap:** Towel wrapped around the neck and held at the back to stabilize the head 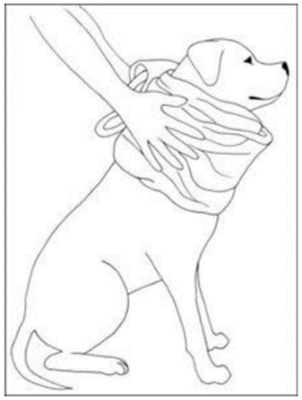	○	○	○	○	○
**Minimal restraint:** Hands are placed on each side of the dog's shoulder allowing some movement of the body and limbs 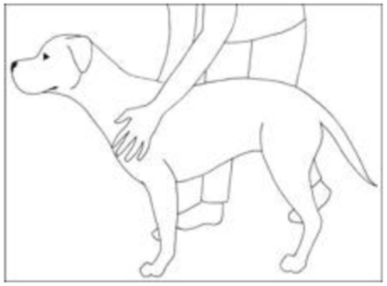	○	○	○	○	○
**Secure restraint:** Hold that secures abdomen with one hand and neck with other hand 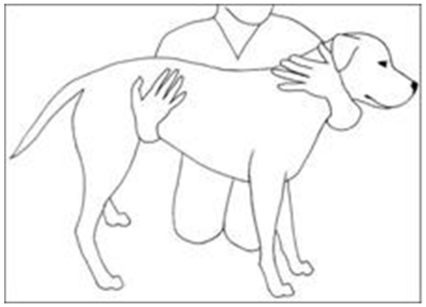	○	○	○	○	○

Head restraint (minimal): Restraint that rests hands on either side of neck with loose hold on collar

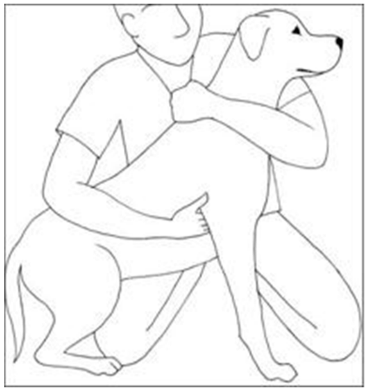

Head restraint (secure): Restraint that secures the neck by pulling head toward handlers' body with one hand and securing side with arm to prevent moving

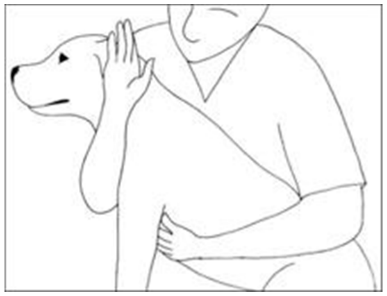

Laying full body restraint: Forward leaning motion with both hands secure around the neck to prevent standing

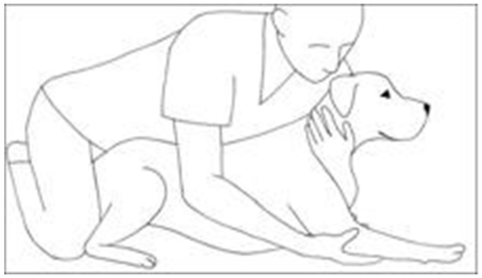

Full body restraint (secure): Restrain all legs using hands while dog is laying on side allowing little to no movement

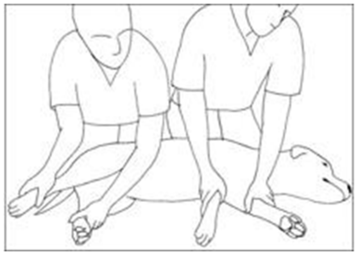

Sitting full body restraint: Restraint that secures the neck and a limb with one hand while in a sitting position to prevent moving back

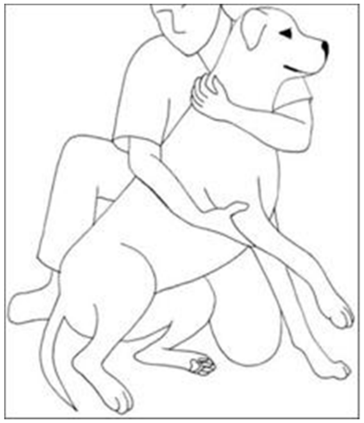

Elizabethan collar: Cone shaped collar that reduces head movement and mouth access beyond collar

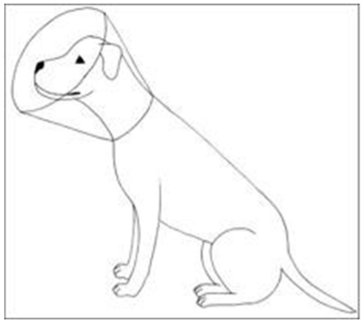

○	○	○	○	○
○	○	○	○	○
○	○	○	○	○

Dog mask: Mask is placed over the eyes and clipped behind the head to reduce visual stimulation

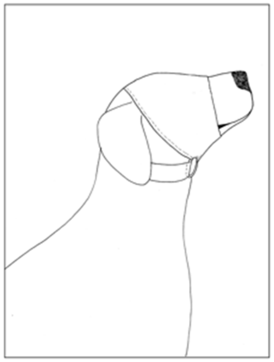

○ ○ ○ ○ ○

Basket muzzle: Muzzle that allows for some mouth movement but limited inside the device

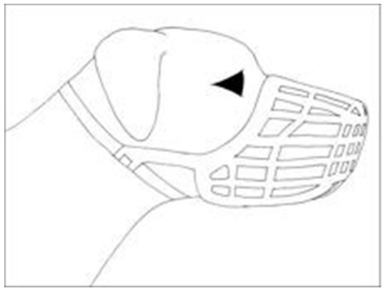

○ ○ ○ ○ ○

13. Please tell us your agreement with the following statements about your dog.

	Strongly Agree	Somewhat Agree	Somewhat Disagree	Strongly Disagree	No Response
My pet means more to me than any of my friends	○	○	○	○	○
Quite often I confide in my pet	○	○	○	○	○
I believe that pets should have the same rights and privileges as family members	○	○	○	○	○
I believe my pet is my best friend	○	○	○	○	○
Quite often, my feelings toward people are affected by the way they react to my pet	○	○	○	○	○
I love my pet because he/she is more loyal to me than most of the people in my life	○	○	○	○	○
I enjoy showing other people pictures of my pet	○	○	○	○	○
I think my pet is just a pet	○	○	○	○	○
I love my pet because it never judges me	○	○	○	○	○
My pet knows when I'm feeling bad	○	○	○	○	○
I often talk to other people about my pet	○	○	○	○	○
My pet understands me	○	○	○	○	○
I believe that loving my pet helps me to stay healthy	○	○	○	○	○
Pets deserve as much respect as humans do	○	○	○	○	○
My pet and I have a very close relationship	○	○	○	○	○
I would do almost anything to take care of my pet	○	○	○	○	○
I play with my pet quite often	○	○	○	○	○
I consider my pet to be a great companion	○	○	○	○	○
My pet makes me feel happy	○	○	○	○	○
I feel that my pet is a part of my family	○	○	○	○	○

I am not very attached to my pet	○	○	○	○	○
Owning a pet adds to my happiness	○	○	○	○	○
I consider my pet to be a friend	○	○	○	○	○

Part III: Owner Demographics

14. Are you:

- ○ Male
- ○ Female
- ○ Non-binary
- ○ Other
- ○ Prefer not to answer

15. Do you have previous experience working in the veterinary field (e.g., veterinarian, veterinary assistant, technician)?

- ○ Yes
- ○ No

16. What is your age (in years)?

- ○ 18-24
- ○ 25-34
- ○ 35-44
- ○ 45-54
- ○ 55-64
- ○ 65-74
- ○ 75 +
- ○ Prefer not to answer

17. Thank you for participating! If you have any comments about the survey, please let us know in the comment box below.

____

____

____

To submit your responses please proceed to the next page.

Thank you for participating!
